# Differential Diagnosis of COVID-19 Pneumonia in Cancer Patients Received Radiotherapy

**DOI:** 10.7150/ijms.46133

**Published:** 2020-09-16

**Authors:** Qi Zeng, Caihua Tang, Lisi Deng, Sheng Li, Jiani Liu, Siyang Wang, Hong Shan

**Affiliations:** 1 Guangdong Provincial Key Laboratory of Biomedical Imaging and Guangdong Provincial Engineering Research Center of Molecular Imaging, The Fifth Affiliated Hospital, Sun Yat-sen University, Zhuhai, Guangdong Province, China, 519000.; 2 Cancer Center,The Fifth Affiliated Hospital, Sun Yat-Sen University, No. 52 Meihua East Road, Zhuhai, Guangdong Province, China, 519000;; 3 Department of Radiology, The Fifth Affiliated Hospital, Sun Yat-Sen University, No. 52 Meihua East Road, Zhuhai, Guangdong Province, China, 519000;; 4 Department of infectious disease, The Fifth Affiliated Hospital, Sun Yat-Sen University, No. 52 Meihua East Road, Zhuhai, Guangdong Province, China, 519000;; 5 Department of Radiology, Sun Yat-Sen University Cancer Center, No. 651 Dongfeng East Road, Guangzhou, Guangdong Province, China, 50060.

**Keywords:** COVID-19, Pneumonia, Radiation pneumonitis, Multidetector computed tomography.

## Abstract

**Background:** During the outbreak period of COVID-19 pneumonia, cancer patients have been neglected and in greater danger. Furthermore, the differential diagnosis between COVID-19 pneumonia and radiation pneumonitis in cancer patients remains a challenge. This study determined their clinical presentations and radiological features in order to early diagnose and separate COVID-19 pneumonia from radiation pneumonitis patients promptly.** Methods and Findings**: From January 21, 2020 to February 18, 2020, 112 patients diagnosed with suspected COVID-19 were selected consecutively. A retrospective analysis including all patients' presenting was performed. Four patients from 112 suspected individals were selected, including 2 males and 2 females with a median age of 54 years (range 39-64 years). After repeated pharyngeal swab nucleic acid tests, 1 case was confirmed and 3 cases were excluded from COVID-19 pneumonia. Despite the comparable morphologic characteristics of lung CT imaging, the location, extent, and distribution of lung lesions between COVID-19 pneumonia and radiation pneumonitis differed significantly. **Conclusions:** Lung CT imaging combined with clinical and laboratory findings can facilitate early diagnosis and appropriate management of COVID-19 pneumonia with a history of malignancy and radiation therapy.

## Introduction

As we were writing this manuscript, severe acute respiratory syndrome coronavirus 2 (SARS-CoV-2) has spread across the world at an alarming rate and become a pandemic^1^. More recent attention has focused on the diagnosis and treatment strategies of coronavirus disease 2019 (COVID-19) caused by a virus belonging to the Coronaviridae family which raises concerns of widespread panic and increases anxiety^2^. Compared to general populations, COVID-19 is more serious and fatal for elderly people and those with underlying physical illnesses and serious mental illnesses^3^. Ever-growing infected and suspected individuals were bound to be isolated, and a large number of medical personnel from other departments such as surgery, oncology, and medicines have been transferred to the frontline departments for coping with the disease^4^. Thus, treatment implementation for patients with malignant tumor has been delayed due to the scarcity of sickbeds and shortage of medical staff in oncology. Unlike ordinary patients, cancer patients are susceptible to infection because of their systemic immunosuppressive state caused by cancer and related treatments, such as chemotherapy and/or radiation therapy (RT). In a recent study, Liang et al. (2020) examined 18 cancer patients in SARS-COV-2 infection, and argued that cancer patients might have a higher risk of COVID-19 and poorer outcomes than individuals without cancer. They also proposed that more intensive surveillance or treatment should be considered when patients with cancer are infected with SARS-CoV-2^5^.

In our fever clinics, of particular concern is the differential diagnosis between radiation-induced lung injury (RILI) and radiological suspicion of COVID-19 pneumonia in patients with malignancy and a history of lung exposure to ionizing radiation. However, there is little discussion about the similarity and the difference between COVID-19 pneumonia and RILI. Therefore, our current study was performed to retrospectively summarize and distinguish their clinical presentations and CT manifestations in order to speed up early diagnosis and patient isolation, and prompt treatment of COVID-19 pneumonia.

## Materials and methods

### Patients

From January 21, 2020 to February 18, 2020, records for patients diagnosed with suspected COVID-19 pneumonia were reviewed retrospectively in our hospital which is the major tertiary teaching hospital in Zhuhai (Guangdong Province, China) and responsible for the treatments for COVID-19 designated by local healthcare authorities. The patients were selected consecutively and met the following criteria: (i) presumed COVID-19 pneumonia according to the diagnostic criteria (version 5) by the National Health Commission of the People's Republic of China. (ii) patients with a history of malignancy and lung exposure to ionizing radiation. No exclusion criteria were applied. This study was approved by the Hospital Review Board and the Medical Ethics Committee, China. We were granted a waiver of written informed consent because it was a retrospective study involved no potential risk to patients. To avoid any potential breach of confidentiality, no link between the researchers and the patients was made available. All patients were evaluated by the following examinations within 2 days after admission: complete patient history, clinical symptoms, physical examination, hematology inspection such as routine blood test, blood biochemistry, arterial blood gas analysis, and detection of T lymphocyte subsets, chest CT, pathogenic examination including nose and pharyngeal swab nucleic acid test for COVID-19, influenza A and B test. If necessary, blood cultures, sputum cultures, and high throughput screening were also performed. All patients received follow-up chest CT after treatment. The management of these patients included isolation, diagnosis, and treatment according to the guideline of COVID-19 (Version 5).

### Chest CT

The detailed protocol has been described in our previous study^6^. Simplely, chest CT scans were performed using 1-mm slice thickness on a UCT 760 scanner (United Imaging; Shanghai, China). To minimize motion artifacts, patients in the supine position were instructed on breath-holding, and CT images were then acquired during end-inspiration without intravenous contrast.

### Image interpretation

All CT images were reviewed by thoracic radiologists and oncologists (QZ, CT and HS with over 5 years of experience) independently and resolved discrepancies by consensus. No negative control cases were examined and no blinding occurred. The axial CT and multiplanar reconstruction images were assessed independently and freely on both lung (width, 1400 HU; level, -500 HU) and mediastinal (width, 350 HU; level, 40 HU) settings to measure ground-glass opacities, consolidation, number of lobes affected by ground-glass or consolidative opacities, degree of lobe involvement, nodules, a pleural effusion, thoracic lymphadenopathy (defined as lymph node size of ≥10 mm in short-axis dimension), underlying lung diseases such as emphysema, fibrosis, cavitation, interlobular septal thickening, reticulation, bronchiectasis, or calcification. The detailed definitions of above CT demonstrations were described as the peer-reviewed literature on COVID-19 pneumonia^6^,^7^. The distribution of lung lesions was documented as predominantly diffuse (continuous involvement without respect to lung segments), subpleural (involving mainly the peripheral one-third of the lung), and cross-segment (confined to radiation fields and nonconformity to anatomic boundaries)^8^.

Previous and follow-up chest CT scans were also reviewed by two radiologists (CTand SL) to evaluate the evolution of lung lesions rated as no significant change, improvement, or progression. Decisions were reached by consensus.

## Results

### Selection of patients with a history of malignancy and radiation therapy

**Figure 1:**
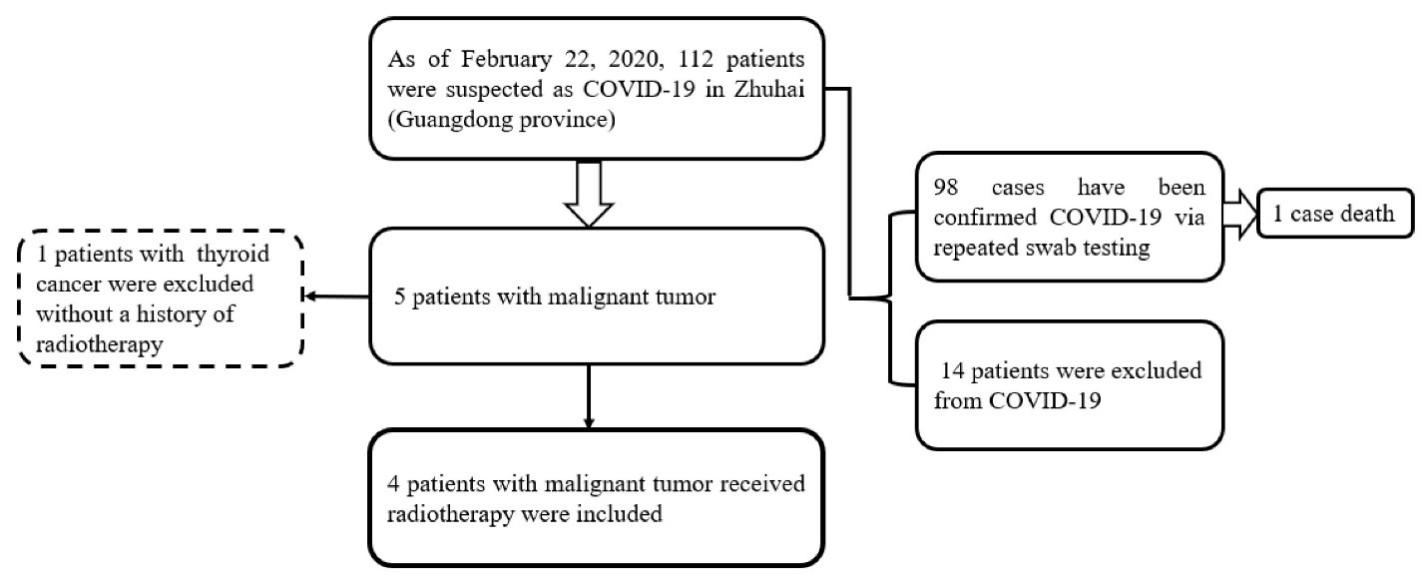
Flowchart of patients

From January 21, 2020 to February 18, 2020, 112 patients with suspected COVID-19 admitted to our hospital. A total of 98 cases were confirmed COVID-19 via repeated swab testing, and 1 case of them was dead later. Another 14 patients were excluded from COVID-19 (Figure 1). Then, among 5 individuals with a history of malignancy, 4 cases who received radiation therapy were included, and 1 case without radiation therapy was excluded from the present study (Figure 1). The median age of 4 cases (2 males and 2 females) was 54 years (range 39-64 years). Two patients with nasopharyngeal carcinoma had completed their concurrent chemoradiotherapy without any signs of tumor recurrence, whereas another 2 patients with advanced thoracic tumors presented unsatisfactory outcomes to anti-tumor systematic therapies including palliative chemotherapy, radiotherapy, or target therapy.

### Confirmation of COVID-19 pneumonia patients with malignancy and radiation therapy

**Figure 2:**
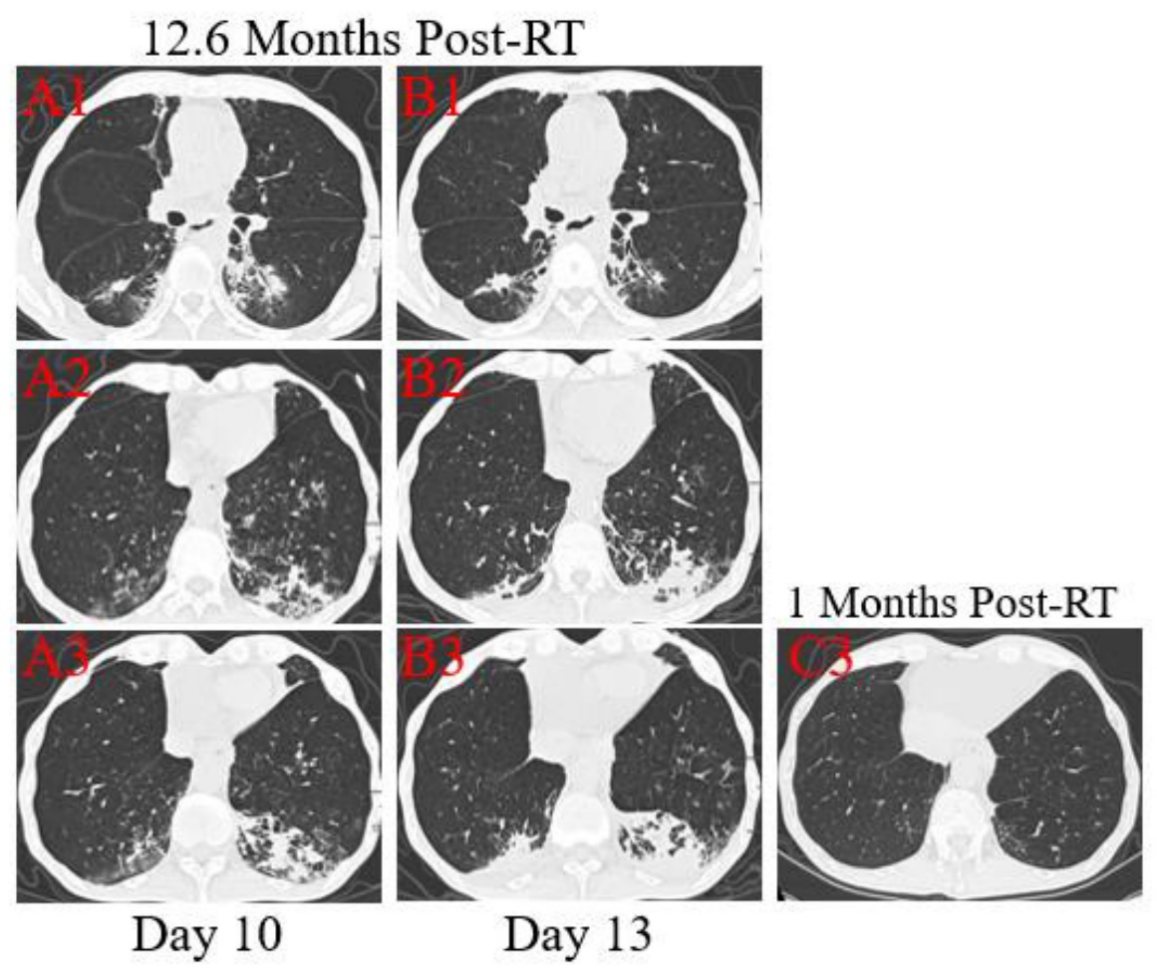
** Transverse thin-section serial CT scans from a 53-year-old male with suspected COVID-19 pneumonia**. Serial CT scans showed pericardial effusion, multiple enlarged lymph nodes in the mediastinum, scattered, multiple, similar round thin wall/no wall transparent areas (A2, B2, C3), smooth or nodular interlobular septal thickening (A1, B1), and multiple nodules in the dorsal segment of the lower lobe of both lungs with spotted calcifications and adjacent pleural thickening (A2, A3). Chest CT images performed at the 10th day after symptom onset showed patchy areas of consolidation co-existed with ground-glass opacities (A3), or linear scarring with discrete consolidation (A2), air bronchograms (A1), and irregular intralobular or interlobular septal thickening (A1-3, A3). Follow-up CT at the 13rd day demonstrated partial improvement (B1) but primarily increment in the extent and density (B2, B3), continued segmental consolidations and atelectasis in the lower lobe of both lungs (B3).

Clinical characteristics (Table 1) and laboratory analysis results (Table 2) of 4 suspected COVID-19 patients with malignancy and radiation therapy at admission were included, and the detailed descriptions of these patients were provided as follows:

**Patient 1**: A 53-year-old male was diagnosed with middle and lower esophageal squamous cell carcinoma with multiple bone metastases staged with T4aN2M1 and began to receive palliative concurrent chemoradiotherapy on September 29, 2016, and admitted to the hospital on January 28, 2020. He had sputum production and cough of more than ten days duration, and a little bit hemoptysis of two days duration. He also felt fatigued, chest distress, vomiting after eating, but no fever. He was neither from the infected area nor contacted with infected peoples. The physical examination revealed coarse breath sounds during auscultation, and laboratory studies showed normal leukocyte, but lymphopenia and serious thrombocytopenia. Also, markly elevated concentrations of D-dimer, Procalcitonin (PCT), C-reactive protein (CRP), and N-terminal-pro hormone brain-type natriuretic peptide (NT-BNP) were observed at admission. Results of serial CT scans showed pericardial effusion, multiple enlarged lymph nodes in the mediastinum, scattered, multiple, similar round thin wall/no wall transparent areas (Figure 2. A2, B2, C3), smooth or nodular interlobular septal thickening (Figure 2. A1, B1), and multiple nodules in the dorsal segment of the lower lobe of both lungs with spotted calcifications and adjacent pleural thickening (Figure 2. A2, A3). Above lung lesions were approximately the same as before. Moreover, compared with the previous CT scan 12.6 months before, chest CT images performed at the 10th day after symptom onset showed the obviously progressive lung lesions including patchy areas of consolidation co-existed with ground-glass opacities (Figure 2. A3), or linear scarring with discrete consolidation (Figure 2. A2), air bronchograms (Figure 2. A1), and irregular intralobular or interlobular septal thickening (Figure 2. A1-3) in the lower lobes of both lungs adjacent to the mediastinum conforming completely to the irradiated area. It was suggested the possibility of RILI, interstitial pneumonia or viral pneumonia. After 3 days of anti-infective therapy with tazocin, moxifloxacin, and arbidol, combined with aggressive supportive care, follow-up CT demonstrated partial improvement (Figure 2. B1) but primarily increment in the extent and density of lung lesions (Figure 2. B2, B3), and continued segmental consolidations and atelectasis were observed in the lower lobe of both lungs (Figure 2. B3). Repeated three times of swab nucleic acid tests for the COVID-19 were negative, and he ultimately was excluded. Afterward, the patient was transferred to the Department of Oncology to continue treatment to reduce the burden of the frontier department.

**Figure 3:**
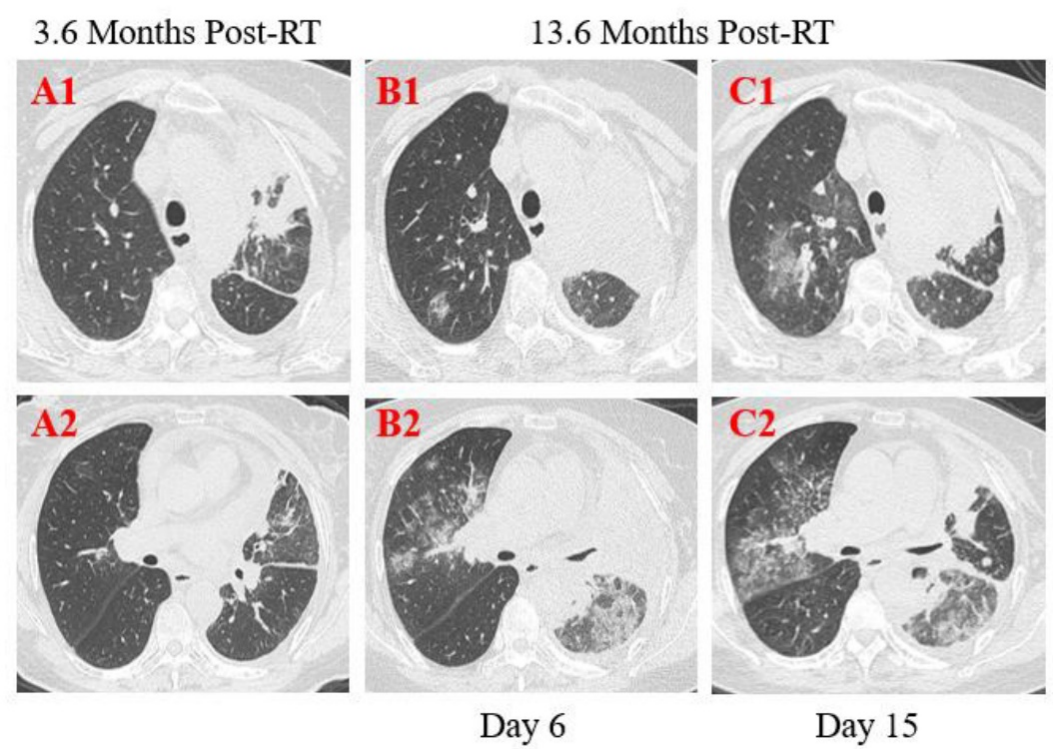
**Transverse thin-section serial CT scans from a 55-year-old female with suspected COVID-19 pneumonia**. Chest CT images performed on the 6th day after symptom onset indicated an enlarged mass with calcification in the left upper lobe and lung hilum and multiple mediastinal lymph node metastases (A1, B1, C1), bilateral diffused ground-glass opacities with partial consolidation (B2). Follow-up CT on the 15th day demonstrated continuous development in the scope and extent of lung lesions (C1, C2).

**Patient 2**: A 55-year-old female was diagnosed with left lung adenocarcinoma with intrapulmonary metastases and multiple bone metastases staged with T4N3M1 and received palliative comprehensive treatment based on target therapy of epidermal growth factor receptor (EGFR) inhibitor in April 2018. She was admitted to the hospital on January 23, 2020, with dyspnea for 1 week and exacerbation for 1 day after more than 1-year targeted therapy. Fatigue, chest distress, and sputum production with cough were also presented in this patient. She had no fever, was not from the infected area, and did not contact with infected persons. The physical examination revealed disappeared breath sounds of the left lung during auscultation, and laboratory studies showed slightly elevated white blood cell and neutrophil, but no changes in lymphopenia. Elevated concentrations of D-dimer, CRP and NT-BNP were displayed at admission. Sputum culture examination revealed normal flora growth without Hemophilus influenza or fungal growth. Furthermore, compared with the previous CT scans 10 months ago, chest CT images performed on the 6th day after symptom onset showed enlarged mass with calcification in the left upper lobe and lung hilum with the maximum section of about 79 mm*48mm, and multiple mediastinal lymph node metastases. The boundary between the left upper lobe and lung hilum is obscure with atelectasis. Magnified irregular nodules scattered in both lungs, and metastatic tumors of the left pleura, the left pleural effusion and pericardial effusion increased in the extent and quantity (Figure 3. A1, B1, C1). There were bilateral diffused ground-glass opacities with partial consolidation (Figure 3. B2), and a reticular pattern associated with bronchiectasis and intralobular or interlobular septal thickening (Figure 3. B2), indicating the possibility of viral pneumonia. After 9 days of anti-infective therapy with tazocin, combined with aggressive supportive care and glucocorticoid therapy (methylprednisolone), follow-up CT demonstrated continuous development in the scope and extent of lung lesions (Figure 3. C1, C2). Repeated two times of swab nucleic acid tests for the COVID-19 were negative, and blood high throughput screening for pathogenic microorganisms or viruses was also negative. The patient's disease continued to progress and she died on February 6, 2020 in the Department of Oncology.

**Figure 4:**
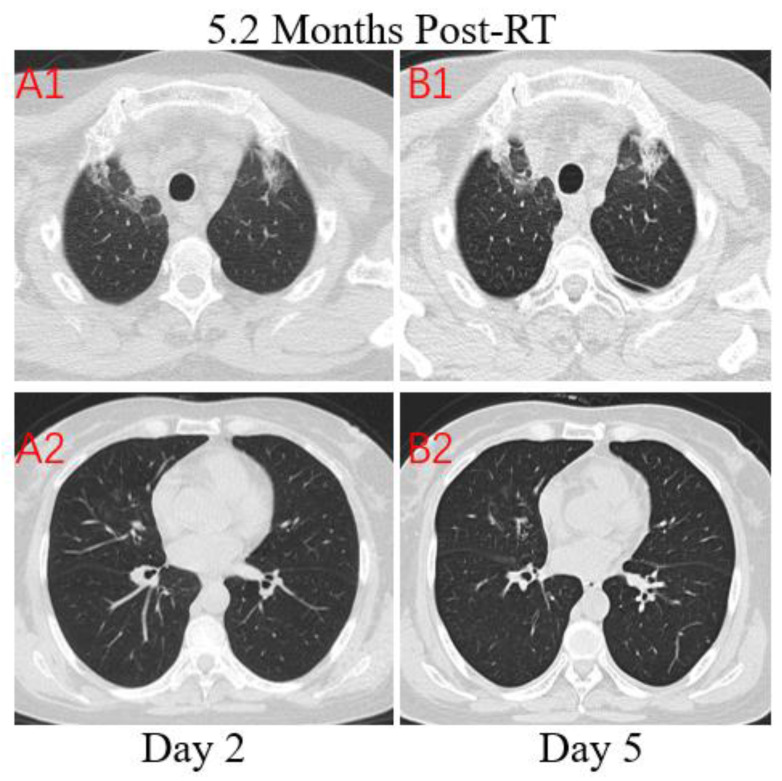
** Transverse unenhanced thin-section serial CT scans from a 64-year-old female with suspected COVID-19 pneumonia**. Chest CT images on the second day after symptom onset found minimal ground-glass opacities with partially rounded consolidation in the apexes of both lungs (A1), and multiple ill-defined patchy ground-glass opacities in the middle lobe of right lung (A2). Follow-up CT on the fifth day demonstrated no obvious change of lung lesions (B1, B2).

**Patient 3:** A 64-year-old woman who worked in Beijing was diagnosed with nasopharyngeal carcinoma(T3N2M0) in July 2019 and treated with definitive concurrent chemoradiotherapy followed by adjuvant chemotherapy. She presented to the hospital with a 1-day history of fever (maximum body temperature was 39.5°C), a little cough and headache on February 17, 2020. The patient traveled to Zhuhai and lived in her community where several patients were confirmed COVID-19. At admission, both lungs were clear on auscultation. Laboratory studies showed normal white blood cell, higher neutrophil, and serious lymphopenia. The concentrations of PCT, CRP, D-dimer and NT-BNP increased significantly. The T lymphocyte subsets test showed a sharp drop in CD4+ and CD8+ T cell counts. Results for influenza A and B antigen screening were negative. Chest CT images were obtained on the second day after symptom onset and indicated that there were minimal ground-glass opacities with partially rounded consolidation (Figure 4. A1) in the apexes of both lungs, conforming completely to the irradiated area of low exposure. Multiple ill-defined patchy ground-glass opacities (Figure 4. A2) were observed in the middle lobe of the right lung, considering the possibility of COVID-19 pneumonia. After 3 days of anti-viral therapy with arbidol, antibiotic treatment with sulperazone, and supportive treatment with albumin injection. Follow-up CT demonstrated no obvious changes of lung lesions (Figure 4. B1, B2). However, the patient's symptoms improved significantly. Repeated four times of swab nucleic acid tests for the COVID-19 were negative. Finally, blood culture suggested an *Escherichia coli* infection. Then the patient was transferred to the Department of Oncology.

**Figure 5:**
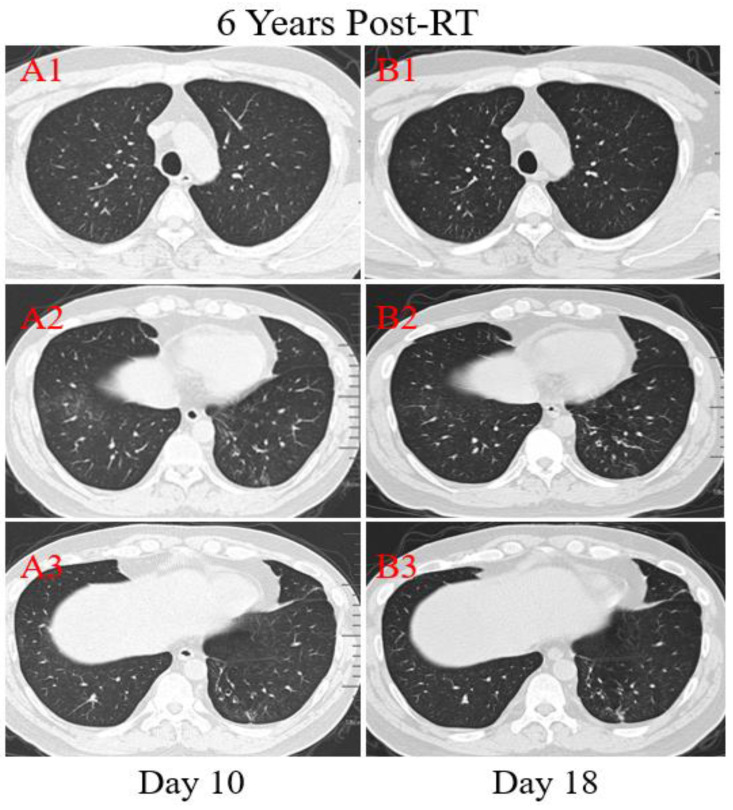
** Transverse unenhanced thin-section serial CT scans from a 39-year-old male with COVID-19 pneumonia**. Chest CT images on the 10th day after symptom onset demonstrated multiple ground-glass opacities of the lower lobes of both lungs peripherally (A2), and a few linear opacities in upper lobe lower lingual segment of the left lung (A3). Follow-up CT at the 18th day demonstrated significant improvement in the extent and density of the ground-glass opacities (B2), and appearance of new focal ground-glass opacities of the upper lobe of right lung (B1).

**Patient 4:** A 39-year-old male was diagnosed with nasopharyngeal carcinoma (T2N2M0) treated with radical concurrent chemoradiotherapy in June 2013. He was admitted to the hospital with a positive result of the swab nucleic acid test for COVID-19 on February 14, 2020. The patient had transient diarrhea 10 days ago but no other symptoms afterward. The patient traveled to Zhuhai from the infected area (Wuhan, China) and had close contact with the confirmed COVID-19 patient, his aunt. At admission, both lungs were clear on auscultation. Laboratory studies showed normal blood routine results. Reslus of influenza A and B antigen screening for this patient were negative. The T lymphocyte subsets test showed a slight drop in CD4+ and CD8+ T cell counts. Chest CT images were obtained on the 10th day after symptom onset and showed that there were multiple ground-glass opacities of the lower lobes of both lungs peripherally and subpleurally (Figure 5. A2). A few linear opacities were presented in upper lobe lower lingual segment of the left lung (Figure 5. A3) within the ionizing radiation area, indicating radiation fibrosis. After 8 days of anti-viral therapy with resochin and supportive treatment, follow-up CT scans demonstrated a significant improvement in the extent and density of the ground-glass opacities (Figure 5. B2), but found a new focal ground-glass opacity (GGO) in the upper lobe of right lung (Figure 5. A1, B1). Treatment continued until the result of the swab test became negative.

## Discussion

Radiation therapy is one of the most common cancer treatment with approximately 50% of all cancer patients receiving radiation therapy during their course of illness, and includes 3D Conformal radiotherapy (3DCRT), intensity modulated radiation therapy (IMRT), image-guided radiotherapy (IGRT), and stereotactic body radiation therapy (SBRT). Especially, radiation therapy remains an important component of treatments for lung cancer, esophageal carcinoma, and nasopharyngeal carcinoma^9^-^12^. RILI is one of the most clinically challenging toxicities secondary to lung radiotherapy or head and neck radiotherapy with an extended field including upper lobes of the lungs^13^. The incidence of RILI is estimated to be 15%-40%^13^. Based on the time interval after the completion of radiotherapy, RILD is typically divided into radiation pneumonitis (RP), occurring within 6 months following radiotherapy (most often within 12 weeks), and pulmonary fibrosis, occurring over 1 year after radiotherapy^14^-^16^. This subject was conducted during my time working for the Department of Infectious Disease. As a radiation oncologist, I was committed to the frontline to deal with COVID-19 due to the shortfall of medical staff from January 23, 2019 to March 5, 2020. During the pandemic period, it was utterly imperative to distinguish COVID-19 pneumonia from other lung pathologies for further isolation and appropriate treatment as early as possible. In this study, the differential diagnosis between RILI and COVID-19 pneumonia in patients with malignancy and a history of lung exposure to ionizing radiation was performed.

The severity of RILI varies from CT imaging abnormalities with no obvious symptoms to life-threatening diseases. Ordinarily, the classic symptoms of RILI include low-grade fevers, non-productive cough, dyspnea on exertion, and hypoxemia^16^. Likewise, previous studies have shown that mild to severe symptoms in COVID-2019 patients included fever, fatigue, dry cough and shortness of breath, and some patients may have dyspnea, productive cough, hemoptysis, myalgia, headache, sore throat, and rhinorrhea^2^,^17^,^18^. These symptoms are particularly difficult to distinguish COVID-2019 from RILI because clinical presentations can be very similar. In our study, three patients with RILI presented with fever, cough, sputum production, dyspnea, or hemoptysis, respectively. By contrast, one patient with confirmed COVID-2019 presented with only transient diarrhea. In a word, no specific symptoms can be definitively used to identify COVID-19.

Laboratory abnormalities are often found in patients with COVID-2019 infection^17^. Laboratory findings in the early stage of COVID-2019 demonstrated normal or higher white blood cells, slight or marked lymphopenia and normal infection-related biomarkers (PCT, CRP), then elevated PCT and CRP in the acute stage of COVID-2019 pneumonia^18^. As the disease progresses, the levels of D-dimer, creatine, creatinine kinase and blood urea are progressively increased before death^19^. Moreover, due to the impact of the tumor itself and treatment-related factors, laboratory tests of patients with RIDI may show signs of inflammation, such as an increased white blood cell, marked lymphopenia, CRP and PCT^14^. The results in our study seem to be coherent with those of previous researches. In addition, it is noteworthy that time course is important for laboratory tests, such as elevated PCT, CRP and D-dimer level always appears in malignancies, RP or bacteria infective pneumonia, whereas no obvious changes are observed in the early stage of COVID-2019^20^. Further laboratory studies are warranted to evaluate for alternate etiologies, including screening for influenza A and B, blood cultures, and the swab nucleic acid tests. Nevertheless, blood cultures and swab tests are a litter bit slow and cumbersome, and the previous study showed that the sensitivity of chest CT for COVID-19 is higher compared to swab test sensitivity (98% VS. 71%, p<.001) for insufficient cellular material and improper extraction of nucleic acid from clinical materials^21^,^22^.

As mentioned above, chest CT scans keep a vital component in the diagnostic algorithm for patients with presumed COVID-19 pneumonia. A recent study suggested that the chest CT images of patients with COVID-19 have certain characteristics with dynamic changes, which are of value for monitoring disease progress and clinical treatment23. The previous researches^5^,^20^,^24^ have established that typical chest CT imaging abnormalities of COVID-19 pneumonia showed unilateral, multifocal, predominantly GGO in the incubation period, with lesions mainly located peripherally and subpleurally, followed by the rapid development of bilateral, diffuse disease in the acute stage, with GGO progressed to or co-existed with consolidations. Subsequently, consolidation continued to increase with GGO, and crazy paving pattern, air bronchograms and irregular intralobular or interlobular septal thickening appeared progressively. These interstitial changes indicated the development of fibrosis. Other findings included pleural effusion, lymphadenopathy, and round cystic changes^20^. However, none of the imaging characteristics of COVID-19 pneumonia seem specific and diagnostic, which bear some resemblance to those of other viral infections and non-infectious conditions. In this study, there are a number of similarities of CT findings between COVID-19 pneumonia and RILI, but serial chest CT imaging of patients can help to continuously monitor the progression or improvement of lung lesions during treatment, sensitively reflecting the differences in COVID-19 pneumonia and RILI. For instance, radiation pneumonitis tends to present on serial lung CT scans as GGO with partial consolidation within 6 months after the completion of irradiation, and evolves into fibrosis in the late stage, including linear scarring with discrete consolidation, air bronchograms and irregular intralobular or interlobular septal thickening. Lung lesions are usually considered to develop slowly and confinement to radiation fields and nonconformity to anatomic boundaries. Accordingly, typical chest CT imaging abnormalities of COVID-19 pneumonia shows unilateral, multifocal, predominantly ground-glass opacities in the early stage, with lesions mainly located peripherally and subpleurally, followed by the rapid development of the bilateral, diffuse disease, with GGO progressed to or co-existed with consolidations within 1-3 weeks after initial symptoms, and finally, crazy paving pattern, air bronchograms and irregular intralobular or interlobular septal thickening appear progressively. Overall, in spite of the comparable morphologic characteristics of lung CT imaging, the location, extent, and distribution of lung lesions between COVID-19 pneumonia and radiation pneumonitis differ significantly.

To the best of our current knowledge, this is the first study that regarding differential diagnosis between COVID-19 pneumonia and RILI. Our findings will facilitate the correct and early diagnosis of COVID-19 pneumonia patients with tumers and under radiotherapy. However, there were several limitations to our study. Firstly, this was a retrospective analysis and sample size was samll due to the scarcity of patients with suspected COVID-19 pneumonia and a history of radiotherapy. Secondly, follow-up CT scans were available only two times and long-term radiological follow-up is needed to confirm our results. Thirdly, due to relatively low sensitivity, repeated swab tests for confirmation of COVID-19 pneumonia are needed. Finally, bacterial pneumonia may be present in some patients in spite of the sputum cultures.

## Conclusions

In summary, there are many similarities of clinical symptoms, laboratory findings and CT imaging features between COVID-19 pneumonia and RILI, in particular radiation pneumonitis. Undoubtedly, elucidating key differences between COVID-19 pneumonia and radiation pneumonitis through a comprehensive evaluation of imaging characteristics combined with clinical and laboratory findings can facilitate early diagnosis and appropriate management.

## Figures and Tables

**Table 1:** Clinical characteristics of 4 patients with tumors and under radiotherapy at admission.

Characteristics	Patient 1	Patient 2	Patient 3	Patient 4
Age, years	53	55	64	39
Sex	Male	Female	Female	Male
Histopathology	Esophageal SCC	Lung adenocarcinoma	NPC	NPC
TNM stage	T4aN2M1	T4N3M1	T3N2M0	T2N2M0
Exposure history	N	N	Y	Y
Comorbidities	Gastric ulcer	N	Sicca syndrome	Hyperthyreosis
PS	3	3	1	0
Symptoms				
Fever	-	-	+	-
Maximum temperature, ℃	-	-	39.5	-
Fatigue	+	+	-	-
Cough	+	+	+	-
Sputum	+	+	-	-
Chest distress	+	+	-	-
Myalgia	-	-	-	-
Dyspnea	-	+	-	-
Hemoptysis	+	-	-	-
Diarrhea	-	-	-	+
Sore throat	-	-	-	-
Vomiting after eating	+	-	-	-
Headache	-	-	+	-
TIME1(days)	10	6	2	10
TIME2(months)	12.6	13.6	5.2	77.9
Treatment	Tazocin+Moxifloxacin +Arbidol	Tazocin+ Methylprednisolone	Sulperazone+Arbidol +Thymosin α1+ Albumin injection	Resochin
Outcomes	Transferred and discharged	Transferred and death	Transferred and discharged	Discharged

**Note**: SCC: squamous cell carcinoma; NPC: nasopharyngeal carcinoma; PS: performance score; TIME1: The time interval between the onset of initial symptoms and the first CT scan at admission; TIME2: The period between the onset of initial symptoms and the first CT scan at admission; Tazocin: Piperacillin-Tazobactam.

**Table 2:** Laboratory findings at admission.

Parameter, unit, (normal value)	Patient 1	Patient 2	Patient 3	Patient 4
WBC, ×10^9^/L, (3.5-9.5)	6.37	10.8	7.87	6.42
Neutrophil, ×10^9^/L, (1.8-6.3)	5.22	9.51	7.25	3.99
Hemoglobin/L, g/L, (130-175)	124	138	119o	144
Lymphocyte, ×10^9^/L, (1.1-3.2)	0.43	0.73	0.10	1.96
Platelet, ×10^9^/L, (125-350)	43	338	96	270
PT, s, (9.4-12.5)	15.40	12	11.90	11.30
APTT, s, (25.1-36.5)	27.60	26.80	30.30	31.80
INR, (0.8-1.15)	1.34	1.11	1.03	0.98
D-dimer, mg/L, (0-243)	1120	11535	482	46
CK, U/L, (39-308)	183	67	29	141
CK-MB, U/L, (0-25)	20.10	2.6	8.10	6.4
LDH, U/L, (120-250)	259	721	153	153
ALT, U/L, (9-50)	11.80	28.10	34.60	26.3
AST, U/L, (15-40)	34.50	34.70	36.30	25.6
Total bilirubin, μmol/L, (3-24)	41.44	4.7	10.10	5.42
BUN, mmol/L, (3.1-8.0)	10.50	5.7	6.3	2.9
Creatinine, μmol/L, (57-111)	82.80	65	90.30	74.9
CTNI, μg/mL, (0-0.0229)	<0.01	<0.01	<0.01	<0.01
NT-BNP, pg/ml, (0-125)	2160	641	208	29
PCT, ng/mL, (0-0.5)	9.09	<0.10	4.62	<0.10
CRP, mg/L, (0.068-8.2)	202.78	88.79	136.56	<0.26
T lymphocyte subsets test				
CD4+T cell, (550-1440)	NR	NR	13	342
CD8+T cell, (320-1250)	NR	NR	39	245
CD4+/CD8+, (0.71-2.78)	NR	NR	0.69	1.4
pathogenic examination	NR	Sputum cultures (-); Blood high throughput screening (-)	Escherichia coli (+) in blood culture; influenza A and B (-)	Influenza A and B (-)
Swab nucleic acid tests of SARS-COV-2 *	Negative (3)	Negative (2)	Negative (5)	Positive

**Note**: WBC: white blood cell; INR: International Normalized Ratio; PT: Prothrombin time; APTT: Activated partial thromboplastin time; CK: Creatine kinase; LDH: Lactate dehydrogenase; ALT: Alanine aminotransferase; AST: Aspartate aminotransferase; BUN: Blood urea nitrogen; CTNI: Troponin I; NT-BNP: N-terminal-pro hormone brain-type natriuretic peptide; PCT: Procalcitonin; CRP: C-reactive Protein; NR: no report. ^*^The numbers in the brackets represent the number times of swab tests.
